# Path Driven Dual Arm Mobile Co-Manipulation Architecture for Large Part Manipulation in Industrial Environments

**DOI:** 10.3390/s21196620

**Published:** 2021-10-05

**Authors:** Aitor Ibarguren, Paul Daelman

**Affiliations:** Industry and Transport Division, TECNALIA, Basque Research and Technology Alliance (BRTA), 20009 San Sebastián, Spain; paul.daelman@tecnalia.com

**Keywords:** mobile co-manipulation, force control, human-robot interaction, robotic application, assistant robots

## Abstract

Collaborative part transportation is an interesting application as many industrial sectors require moving large parts among different areas of the workshops, using a large amount of the workforce on this tasks. Even so, the implementation of such kinds of robotic solutions raises technical challenges like force-based control or robot-to-human feedback. This paper presents a path-driven mobile co-manipulation architecture, proposing an algorithm that deals with all the steps of collaborative part transportation. Starting from the generation of force-based twist commands, continuing with the path management for the definition of safe and collaborative areas, and finishing with the feedback provided to the system users, the proposed approach allows creating collaborative lanes for the conveyance of large components. The implemented solution and performed tests show the suitability of the proposed architecture, allowing the creation of a functional robotic system able to assist operators transporting large parts on workshops.

## 1. Introduction

The emergence of collaborative robotics is changing the way of developing new robotic applications, especially in those cases involving cooperative tasks between humans and robots. This kind of cooperative task raises many technical challenges, ranging from the use of force feedback to guide the collaborative process [1] to the analysis of the social and psychological aspects of the acceptance of these new technologies [2].

Robotic part transportation is a compelling application as many industrial sectors such as the aeronautic [3,4] or automotive [5,6] require moving large parts among different areas of the workshops, using a large amount of the workforce on tasks with no added value. Therefore, the design and development of collaborative solutions based on flexible robotic systems able to transport large pieces [7,8,9] would benefit a wide range of companies worldwide. However, these systems need to deal with multiple technical topics like the management of the force feedback to generate movement commands or the definition of collaborative areas within the workshops where the robots can move freely.

This paper presents a path-driven dual-arm co-manipulation architecture for large part transportation. This architecture addresses three key aspects of the collaborative part transportation task: (1) Human-driven mobile co-manipulation, (2) soft superposition of navigation trajectories to the co-manipulation task to ensure safety zones within the workshop, and (3) robot-to-human feedback to guide and facilitate the collaborative task. The architecture tackles these three topics, proposing a new scheme that addresses issues for the industrial implementation of this kind of systems like safety and real-time feedback of the process state. The implementation and evaluation of the architecture demonstrate its suitability for cooperative applications in industrial environments.

The paper is organized as follows. Section 2 provides information about related work. Section 3 presents the proposed architecture, including the different modules of the approach. Details about the implementation of the architecture are given in Section 4. Section 5 provides further information about the experiments carried out to test the suitability of the proposed architecture. Finally, Section 6 contains information about the conclusions and future work.

## 2. Related Work

Human-robot manipulation is a broad research topic, with multiple approaches proposed for a wide range of scenarios and applications. From classical scenarios with industrial robotic manipulators [10], to the use of new humanoid robots [11], many papers deal with the co-manipulation topic. From the industrial perspective, mobile manipulators offer an appealing and flexible solution as they allow extending manipulation capabilities of robots along the whole production area. As an example, Engemann et al. [12] present an autonomous mobile manipulator for flexible production, which includes a novel workspace monitoring system to ensure safe human-robot collaboration.

Force control is one of the most studied fields in human-robot collaboration, with a wide range of algorithms and approaches to offer a seamless interaction based on force. Peternel et al. [13] propose an approach for co-manipulation tasks such as sawing or bolt screwing through a human-in-the-loop framework, integrating online information about manipulability properties. Lichiardopol et al. [1] pose a control scheme for human-robot co-manipulation with a single robot, a system able to estimate an unknown and time-varying mass and the force applied by the operator. Focusing on mobile manipulators, Weyrer et al. [14] present a natural approach for hand guiding a sensitive mobile manipulator in task space using a force-torque sensor.

Additionally, the use of guides and predefined trajectories for dual-arm and co-manipulation tasks appear in different research activities. Gan et al. [15] present a position/force coordination control for multi-robot systems where an object-oriented hierarchical trajectory planning is adopted as the first step of a welding task. Jlassi et al. [16] introduce a modified impedance control method for heavy load co-manipulation; an event-controlled online trajectory generator is included to translate the human operator intentions into ideal trajectories. Continuing with the topic of trajectory generation, Raiola et al. [17] propose a framework to design virtual guides through demonstration using Gaussian mixture models. The use of these paths and guides are interesting concepts that could be transferred to mobile co-manipulation.

Multi-robot systems are an alternative for force-based large part co-manipulation. Hichri et al. address the flexible co-manipulation and transportation task with a mobile multi-robot system, tackling issues like the mechanical design [7] and optimal positioning of a group of mobile robots [18]. However, these approaches leave human operators out of the control strategy.

The use of Artificial Intelligence for mobile manipulation and cooperative tasks is also a recurrent research topic, as it adds mechanisms to tune and optimize control parameters. Zhou et al. [19] propose a mobile manipulation method integrating deep-learning-based multiple-object detection to track and grasp dynamic objects efficiently. Following this same path, Wang et al. [20] present a novel mobile manipulation system that decouples visual perception from the deep reinforcement learning control, improving its generalization from simulation training to real-world testing. Additionally, Iriondo et al. [21] include a deep reinforcement learning (DRL) approach for pick and place operations in logistics using a mobile manipulator. Moreover, deep learning algorithms have also been used in force-based human-robot interaction, specifically for the identification of robot tool dynamics [22], allowing a fast computation and adding noise robustness.

From the user point of view, it is also important to include human and social factors in the design of collaborative applications as the lack of transparency in robot behavior can worsen the user experience, as shown by Sanders et al. and Wortham and Theodorou [23,24]. Therefore, it is crucial to add mechanisms to communicate robot intentions and provide continuous feedback as it helps improving task performance [25] and increasing trust [23]. As an example of these previous words, Weiss et al. [26] present a work where operators use and program collaborative robots in industrial environments in various case studies, providing an additional anthropocentric dimension to the discussion of human-robot cooperation.

The main contribution of the presented work is the usage of navigation paths in force-based mobile co-manipulation. This addition allows modifying the commands generated from the force information and providing mechanisms to limit the robot’s workspace. This feature enables the definition of safety areas within the workshops, a critical issue in the implementation of this kind of application in a production environment.

## 3. Proposed Architecture

Collaborative part transportation is an appealing application, as many industrial sectors such as the aeronautic or automotive one require moving large parts among different areas of the workshops, spending a large quantity of workforce on tasks with no added value. Therefore, it is interesting to offer robotic solutions that allow collaborative transportation, paying attention to different aspects such as the control algorithm or robot-to-human feedback.

In an initial step of the development, the large part transportation process has been analyzed to extract the key elements of the task, as well as the requirements to be transferred to the robotic solution:

- During large part transportation by humans, both actors agree (implicit or explicitly) on an approximate path to follow during the process. Additionally, humans can indicate their destination during the task. Therefore, the robot should include some feedback indicating which direction they are (or should be) moving.
- As robots act as assistants of the human, robots will only advance in the path when the operator moves the part in the defined direction. Therefore, humans always have the master role in the co-manipulation task.
- Industrial workshops generally include lanes used for the transit of vehicles and large machines. The mobile co-manipulation system should offer mechanisms to limit the co-manipulation areas and restrict them to the allowed zones when required. These safety measures are mandatory for the industrial implementation of this kind of solution.

To cope with the previously listed requirements, a four-layer architecture is proposed:

- **Force Management Layer:** This initial layer is in charge of generating twist commands for the mobile robotic platform based on the force information received from the arms. This force information could be provided by force/torque sensors attached to the grippers or directly by robots equipped with internal sensors.
- **Moving Average Filter Layer:** This second layer filters the received twist commands to smooth the velocity.
- **Path Management Layer:** This third layer modifies, if necessary, the smoothed twist commands to ensure that the robotic platform is within the safety lanes defined in the workshop.
- **User Interface Management Layer:** This last layer is in charge of presenting the co-manipulation feedback to operators, using different cues to this end.

**Figure 1:**
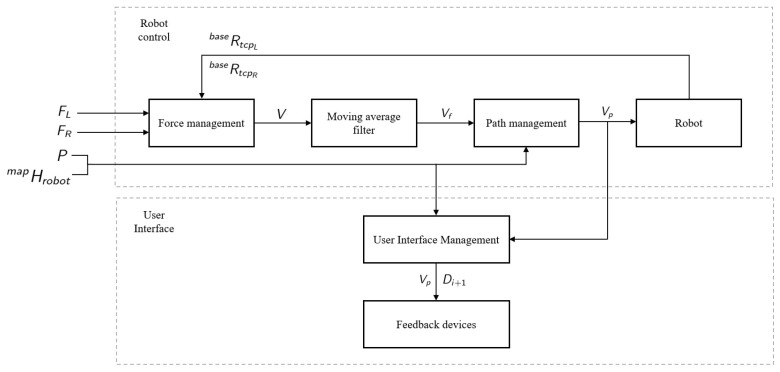
Proposed architecture for mobile part co-manipulation.

This four-layer architecture allows the mobile co-manipulation task, including different modules for the robot control besides the generation of robot-to-human feedback. It ensures that the system controls all the process steps, from low-level control to high-level interaction feedback. Figure 1 illustrates the presented architecture.

The following sections provide further information about the different layers and their specific features.

### 3.1. Force Management Layer

The first step is to estimate the external forces applied on the left arm $F_{e_{L}}$ and right arm $F_{e_{R}}$ in the robot base frame as

(1) $$F_{e_{L}}={\;}^{base}R_{tcp_{L}}\;F_{L}-m_{L}\;G,$$

(2) $$F_{e_{R}}={\;}^{base}R_{tcp_{R}}\;F_{R}-m_{R}\;G,$$

where $F_{L}$ and $F_{R}$ are the forces sensed in the TCP of the left and right arm, ${}^{base}R_{tcp_{L}}$ and ${}^{base}R_{tcp_{R}}$ define the rotation of the left and right arm TCP in robot’s base frame, $m_{L}$ and $m_{R}$ are the mass of the left and right arm tool and *G* is the gravity vector ${\left[0,0,-g\right]}^{T}$.

The external torques applied on the left arm $T_{e_{L}}$ and right arm $T_{e_{R}}$ in the robot base frame are calculated as

(3) $$T_{e_{L}}={\;}^{base}R_{tcp_{L}}\;\left(T_{L}-\;m_{L}r_{L}\times {(}^{base}R_{tcp_{L}}^{-1}\;G)\right),$$

(4) $$T_{e_{R}}={\;}^{base}R_{tcp_{R}}\;\left(T_{R}-\;m_{R}r_{R}\times {(}^{base}R_{tcp_{R}}^{-1}\;G)\right),$$

where ${}^{base}R_{tcp_{L}}$ and ${}^{base}R_{tcp_{R}}$ define the rotation of the left and right arm TCP in robot’s base frame, $T_{L}$ and $T_{R}$ are the torques sensed in the TCP of the left and right arm, $m_{L}$ and $m_{R}$ are the mass of the left and right arm tool, $r_{L}$ and $r_{R}$ are the center of mass of the left and right arm tool and *G* is the gravity vector ${\left[0,0,-g\right]}^{T}$.

The overall external force $F_{e}$ and torque $T_{e}$ are calculated as

(5) $$F_{e}=\left(F_{e_{L}}+F_{e_{R}}\right)/2,$$

(6) $$T_{e}=\left(T_{e_{L}}+T_{e_{R}}\right)/2,$$

using the applied forces of the left arm $F_{e_{L}}$ and right arm $F_{e_{R}}$, as well as the applied torques of the left arm $T_{e_{L}}$ and right arm $T_{e_{R}}$ estimated previously.

These force and torque vectors $F_{e}={\left[f_{e_{X}},f_{e_{Y}},f_{e_{Z}}\right]}^{T}$ and $T_{e}={\left[t_{e_{X}},t_{e_{Y}},t_{e_{Z}}\right]}^{T}$ are used afterwards to calculate the twist vector *V* that will be sent to the mobile platform

(7) $$V={\left[\nu_{X},\nu_{Y},\omega_{Z}\right]}^{T},$$

where $\nu_{X}$ and $\nu_{Y}$ define the linear velocity in axis *X* and *Y*, respectively, while $\omega_{Z}$ indicates the angular velocity in axis *Z*.

In the generation of this twist vector *V*, the idea is to define a force and torque range where the robot will move. If the sensed forces and torques are below a predefined threshold, the twist values will be equal to zero. These twist values will increase linearly as forces and torques increase, defining a maximum allowed force and torque, as well as a maximum robot speed. It will allow defining different force/torque and velocity profiles, profiles that can be used on different phases of the part transportation such as the fast movement between stations of the workshop or the precise and slow positioning of the robotic platform at the part loading area.

The linear velocities $\nu_{X}$ and $\nu_{Y}$ are calculated as

(8) $$\nu_{X}=\left\{\begin{array}{cc} 0, & \mathrm{if}\;\;\;\;f_{e_{X}}<min_{F}\\ max_{\nu }*MIN\left(f_{e_{X}},max_{F}\right)/max_{F}, & \mathrm{otherwise}, \end{array}\right.$$

(9) $$\nu_{Y}=\left\{\begin{array}{cc} 0, & \mathrm{if}\;\;\;\;f_{e_{Y}}<min_{F}\\ max_{\nu }*MIN\left(f_{e_{Y}},max_{F}\right)/max_{F}, & \mathrm{otherwise}, \end{array}\right.$$

where $f_{e_{X}}$ and $f_{e_{Y}}$ are the external forces in axis *X* and *Y*, $min_{F}$ and $max_{F}$ define the minimum required force and the maximum allowed force and $max_{\nu }$ represents the maximum allowed linear velocity.

The angular velocity $\omega_{Z}$ is calculated as

(10) $$\omega_{Z}=\left\{\begin{array}{cc} 0, & \mathrm{if}\;\;\;\;t_{e_{Z}}<min_{T}\\ max_{\omega }*MIN\left(t_{e_{Z}},max_{T}\right)/max_{T}, & \mathrm{otherwise}, \end{array}\right.$$

where $t_{e_{Z}}$ is the external torque in axis *Z*, $min_{T}$ and $max_{T}$ define the minimum required torque and the maximum allowed torque and $max_{\omega }$ represents the maximum allowed angular velocity.

This twist vector $V={\left[\nu_{X},\nu_{Y},\omega_{Z}\right]}^{T}$ is the information sent to the next *Moving Average Filte Layer*.

### 3.2. Moving Average Filter Layer

This layer applies a classical *moving average filter* [27] to the twist vector obtained in the previous layer to ensure a smooth navigation of the mobile platform. The filtered twist vector $V_{f}$ is calculated as

(11) $$V_{f}=\frac{1}{K}\sum_{i=0}^{K}V_{i}\;,$$

where *K* defines the size of the filter and $V_{i}$ represents the last received *K* twist vectors.

This filtered vector $V_{f}=\left[\nu_{f_{X}},\nu_{f_{Y}},\omega_{f_{Z}}\right]$ will be sent to the *Path Management Layer*, layer that will modify it in order to fit in the path provided to the algorithm.

### 3.3. Path Management Layer

**Figure 2:**
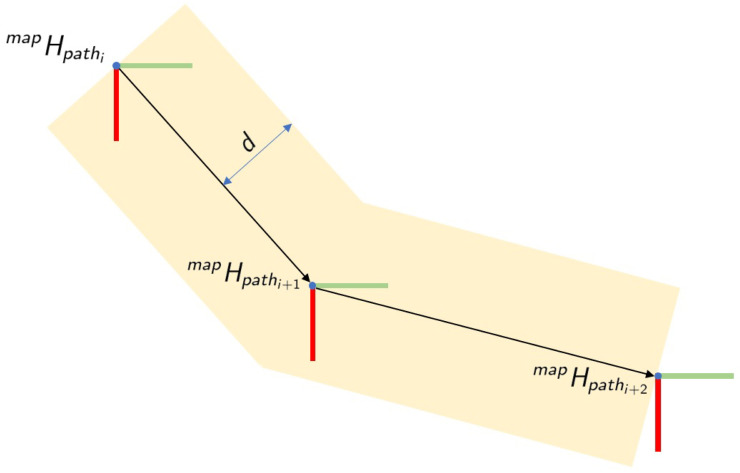
Definition of path.

The main idea of this layer is to define a corridor where the robotic platform can move freely, limiting the movements when the robot is trying to go outside the borders of the lane. It allows the creation of some virtual walls at both sides of the lane, as shown in Figure 2, defining the area where the operator can co-manipulate the part.

Therefore, the filtered twist vector $V_{f}$ is corrected based on the provided path *P* and the current pose of the robotic platform in the map ${}^{map}H_{base}$. Specifically, the path is composed of a list of *M* poses as

(12) $$P={\{}^{map}H_{path_{1}}\;{,}^{map}H_{path_{2}}\;,\ldots {,}^{map}H_{path_{M}}\},$$

where ${}^{map}H_{path_{i}}$ defines the $i^{th}$ pose of the path in the map frame. Additionally, the current pose of the robotic platform ${}^{map}H_{base}$ is defined as

(13) $${}^{map}H_{base}=\left[\begin{array}{c} \begin{array}{cc} {}^{map}R_{base} & {}^{map}T_{base}\\ 0 & 1 \end{array} \end{array}\right],$$

where ${}^{map}R_{base}$ and ${}^{map}T_{base}$ represent the rotation and translation part of the transformation matrix.

**Figure 3:**
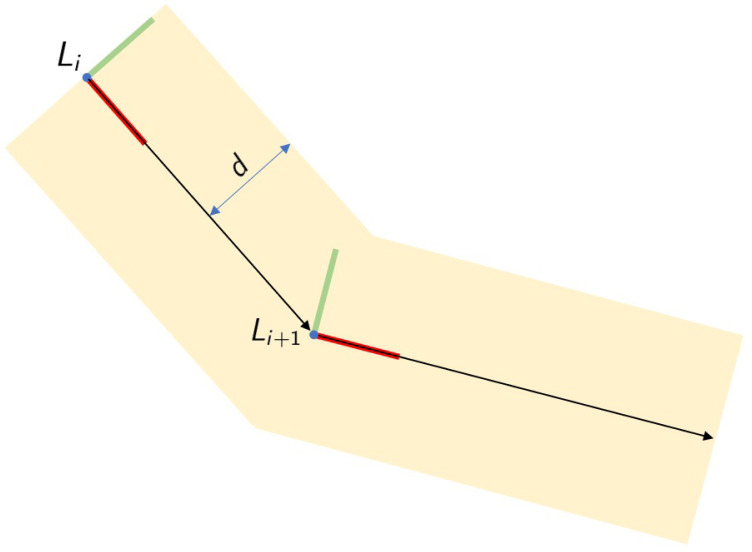
Calculation of path frame L.

In an initial step, a new coordinate system *L* is calculated, a coordinate system with the origin in ${}^{map}T_{path_{i}}$ (translation part of $i^{th}$ path pose) with axis *X* pointing to pose ${}^{map}H_{path_{i+1}}$ (Figure 3). The calculus of this new coordinate system allows for creating a local frame for the current path segment, which facilitates the calculations of the corrections to be applied. The coordinate system *L* is calculated using the following equations:

(14) $$A=\frac{{}^{map}T_{path_{i+1}}-{\;}^{map}T_{path_{i}}}{{|}^{map}T_{path_{i+1}}-{\;}^{map}T_{path_{i}}|}=\left[\begin{array}{c} a_{1}\\ a_{2}\\ a_{3} \end{array}\right],$$

where *A* defines the unitary vector pointing from path pose *i* to pose i+1,

(15) $$Y={(}^{map}T_{path_{i+1}}-{\;}^{map}T_{path_{i}})\times \left[\begin{array}{c} 0\\ 0\\ -1 \end{array}\right],$$

(16) $$C=\frac{A\times Y}{\left|A\times Y\right|}=\left[\begin{array}{c} c_{1}\\ c_{2}\\ c_{3} \end{array}\right],$$

where *C* defines the unitary vector pointing upwards in the map and

(17) $$B=\frac{C\times A}{\left|C\times A\right|}=\left[\begin{array}{c} b_{1}\\ b_{2}\\ b_{3} \end{array}\right],$$

where *B* defines the unitary vector pointing perpendicularly to the lane border.

Based on these three unitary vectors, the new coordinate system is calculated as

(18) $$L=\left[\begin{array}{cc} \begin{array}{cccc} a_{1} & b_{1} & c_{1} & \\ a_{2} & b_{2} & c_{2}\\ a_{3} & b_{3} & c_{3} \end{array} & {}^{map}T_{path_{i}}\\ 0 & 1 \end{array}\right].$$

where unitary vectors *A*, *B*, and *C* define the rotational part of the transformation matrix and vector ${}^{map}T_{path_{i}}$ indicates the translational part of the matrix.

Once this path frame *L* is defined, the poses of the robotic platform ${}^{L}H_{base}$ and next path pose ${}^{L}H_{path_{i+1}}$ in the path frame are calculated as

(19) $${}^{L}H_{base}=L^{-1}\;\cdot {\;}^{map}H_{base}=\left[\begin{array}{c} \begin{array}{cc} {}^{L}R_{base} & {}^{L}T_{base}\\ 0 & 1 \end{array} \end{array}\right],$$

(20) $${}^{L}H_{path_{i+1}}=L^{-1}\;\cdot {\;}^{map}H_{path_{i+1}}=\left[\begin{array}{c} \begin{array}{cc} {}^{L}R_{path_{i+1}} & {}^{L}T_{path_{i+1}}\\ 0 & 1 \end{array} \end{array}\right],$$

where ${}^{L}R_{base}$ and ${}^{L}T_{base}$ represent the rotation and translation part of the robotic platform pose in the path frame and ${}^{L}R_{path_{i+1}}$ and ${}^{L}T_{path_{i+1}}$ represent the rotation and translation part of the next path pose in the path frame.

At this point, the theoretical next robotic platform pose is calculated as

(21) $${}^{L}T_{base}^{t+1}={\;}^{L}H_{base}\;\cdot \left[\begin{array}{c} \nu_{X}\\ \nu_{Y}\\ 0 \end{array}\right]\Delta t=\left[\begin{array}{c} {}^{L}t_{base_{X}}^{t+1}\\ {}^{L}t_{base_{Y}}^{t+1}\\ {}^{L}t_{base_{Z}}^{t+1} \end{array}\right],$$

where ${}^{L}H_{base}$ defines the current robotic platform pose in path frame, $\nu_{X}$ and $\nu_{Y}$ represents the linear twist values in axis *X* and *Y*, and Δt defines the period of the control loop.

In the next step, the corrected next platform pose ${}^{L}T_{base}^{*}$ is calculated

(22) $${}^{L}T_{base}^{*}=\left[\begin{array}{c} {}^{L}t_{base_{X}}^{*}\\ {}^{L}t_{base_{Y}}^{*}\\ {}^{L}t_{base_{Z}}^{*} \end{array}\right],$$

ensuring that the robotic platform does not cross the borders of the defined lane, lane created based on the path *P* and the maximum distance *d* from the path. Specifically, the corrected next platform pose is calculated as

(23) $${}^{L}t_{base_{X}}^{*}=\left\{\begin{array}{cc} {}^{L}t_{base_{X}}^{t+1}\;, & {\mathrm{if}\;\;\;\;|}^{L}t_{base_{X}}^{t+1}|\leq d\\ {}^{L}t_{base_{X}}\;, & \mathrm{otherwise} \end{array}\right.,$$

(24) $${}^{L}t_{base_{Y}}^{*}=\left\{\begin{array}{cc} {}^{L}t_{base_{Y}}^{t+1}\;, & {\mathrm{if}\;\;\;\;|}^{L}t_{base_{Y}}^{t+1}|\leq d\\ {}^{L}t_{base_{Y}}\;, & \mathrm{otherwise} \end{array}\right.,$$

(25) $${}^{L}t_{base_{Z}}^{*}{=}^{L}t_{base_{Z}}^{t+1},$$

where it is ensured that the limits are not crossed in axes *X* and *Y*.

Finally, the corrected twist values $V_{p}$ are calculated as

(26) $$V^{*}=\frac{{}^{L}H_{base}^{-1}\;\cdot {\;}^{L}T_{base}^{*}}{\Delta t}=\left[\begin{array}{c} \nu_{X}^{*}\\ \nu_{Y}^{*}\\ \nu_{Z}^{*} \end{array}\right],$$

(27) $$V_{p}=\left[\begin{array}{c} \nu_{X}^{*}\\ \nu_{Y}^{*}\\ \omega_{Z} \end{array}\right],$$

where ${}^{L}H_{base}$ defines the current pose of the robotic platform in the path frame, ${}^{L}T_{base}^{*}$ represents the corrected next platform pose, Δt sets the period of the control loop, and $\omega_{Z}$ represents the original angular velocity in axis *Z* as it will not be limited nor modified by the *Path Management Layer*.

This new twist vector $V_{p}$ will be sent to the robot for its execution, as it ensures that the robot maintains within the limits defined by the path *P* and the maximum distance *d*. Additionally, this vector $V_{p}$ is also sent to the *User Interface Management Layer* to generate the appropriate feedback for the co-manipulation process.

### 3.4. User Interface Management Layer

This last layer will manage the generated twist commands, besides the current robot status, to provide helpful feedback to operators. Specifically, two different feedback cues have been considered:

- **Movement direction:** The movement direction is directly generated using twist vector $V_{p}$, constantly indicating where the robot is moving.
- **Direction to the next path pose:** The second cue defined to guide the co-manipulation process is the direction vector to the next path pose $D_{i+1}$, which is calculated as
  (28) $$D_{i+1}={\;}^{map}H_{base}^{-1}\;\cdot {\;}^{map}T_{path_{i+1}}=\left[\begin{array}{c} d_{X}\\ d_{Y}\\ d_{Z} \end{array}\right],$$
  where ${}^{map}H_{base}$ is the pose of the robotic platform in the map frame and ${}^{map}T_{path_{i+1}}$ indicates the translation from the map frame to the next path pose.

Additionally, some audio signals have been added to indicate when the path-driven mobile co-manipulation starts and when the destination has been reached. These audio cues would complete the feedback sent from the robot to the user to guide the mobile co-manipulation task.

## 4. Implementation

**Figure 4:**
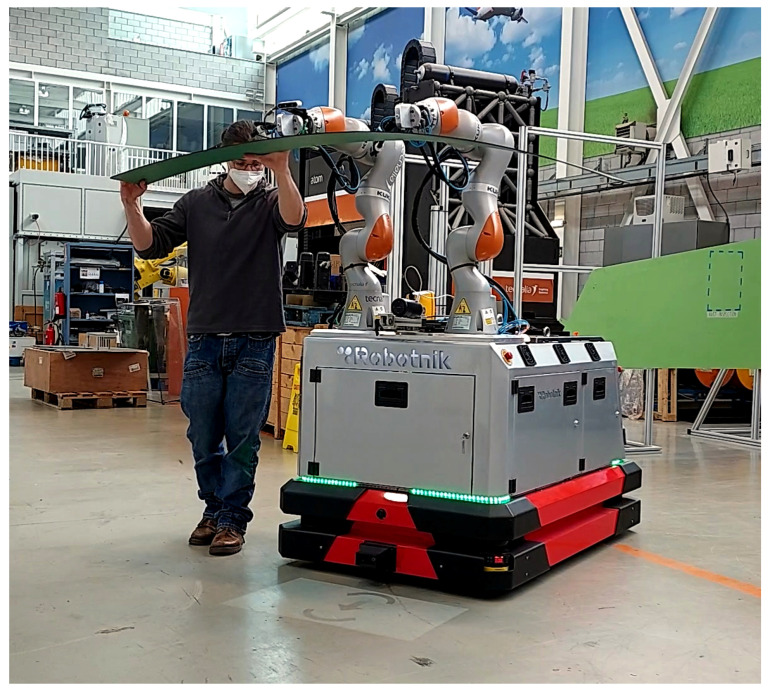
Dual-arm omnidirectional mobile robot.

**Figure 5:**
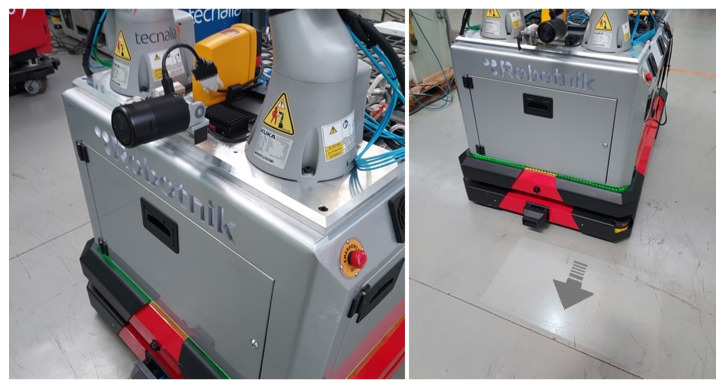
Projector installed on robotic platform to display the direction to the next path pose.

The proposed architecture has been implemented in a dual-arm mobile platform designed for the manipulation of large parts in industrial environments, see Figure 4. The robotic platform is composed of this hardware:

- An omnidirectional mobile platform equipped with Mecanum wheels [28]. The platform includes led lights placed in the front part, rear part, and both sides of the base. These lights are used to provide the feedback of the *movement direction* generated in the *User Interface Management Layer*. Therefore, the robot’s front and rear lights blink when the robot moves forward or backward, while the sidelights blink when the robot moves in these directions.
- Two *Kuka LBR iiwa* robots with a payload of 7 kg, collaborative arms equipped with torque sensors in each joint. These robotic arms can provide force and torque information estimated from the torque sensors placed in each joint. Therefore, this implementation does not require an external sensor to acquire force and torque information.
- The arms are also equipped with automatic tool exchangers, a suction system, and vacuum cups to grasp different types of large objects and parts.
- An additional IO module is also available to manage the tool exchangers and the suction of the vacuum cups.
- A small size projector has been installed in the front part of the robot. This projector is used to display the *direction to the next path pose* on the ground in the front part of the robot as shown in Figure 5.

From the software point of view, a PC placed inside the robotic platform executes the four modules of the architecture. All these modules have been implemented in C++ as ROS nodes. An additional HTML5 server provides the web interface that shows the arrows and cues used to guide the co-manipulation process.

Finally, an HMTL5 based general User Interface (UI) has been developed. This interface allows commanding the system, triggering and canceling the execution of the mobile co-manipulation at any moment. A tablet is used to display this UI, enabling operators to utilize this interface at any place within the workshop.

## 5. Experiments

**Figure 6:**
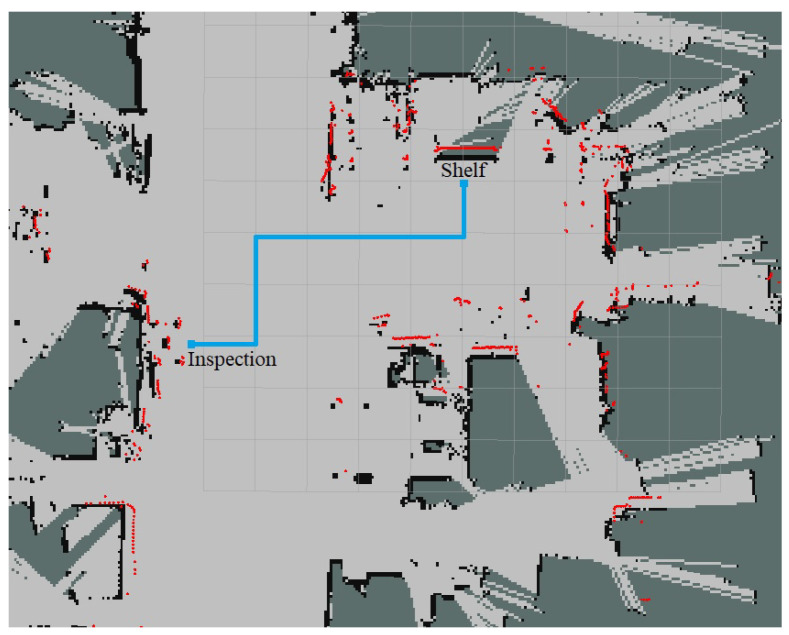
Co-manipulation path on workshop map.

**Figure 7:**
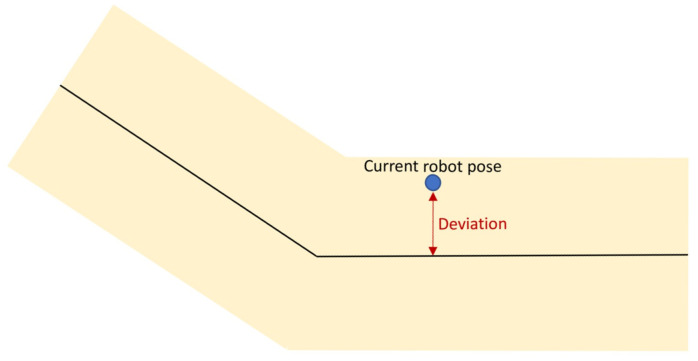
Deviation from the nominal path.

As the last step, the presented architecture has been evaluated through a set of tests. Specifically, an experiment was defined in which various users had to transport a carbon fiber part between two stations placed in an industrial workshop using the mobile co-manipulation architecture presented above. The next lines provide information about the experiment:

- Two different stations were defined in the workshop, *shelf* and *inspection station*, 10 m away from each other (see Figure 6).
- Five different users transported a 2-m carbon fiber part between stations alongside the robot. The subjects did not have any previous knowledge about the defined path.
- To get a better insight into the process, the percentage of the covered trajectory and the deviation from the nominal path were recorded with a frequency of 15 Hz. The deviation from the nominal path indicates the perpendicular distance between the current robot pose and the central line of the path, as shown in Figure 7.
- During the transportation, two different maximum distances from the path (*d*) were used, 0.250 m and 0.500 m, defining lanes with a width of 0.500 m and 1.0 m, respectively. It will serve to assess the impact of the lane width on the trajectory deviation.

**Figure 8:**
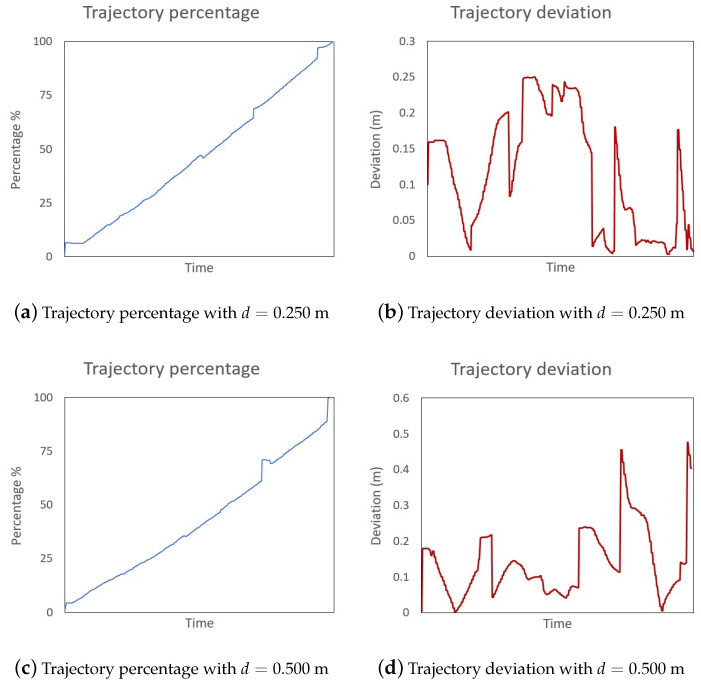
Trajectory percentage and deviation of two transportation processes.

Therefore, each of the five subjects performed four different transportation processes, moving the part in both directions (*shelf to inspection* and *inspection to shelf*) with two different lane widths. Figure 8 shows trajectory percentage and deviation plots extracted from two transportation processes.

Table 1 summarizes the obtained results. The first column indicates the maximum allowed distance from path *d* used during the transportation. The second column includes the route of the transportation. The third and fourth columns provide information about the mean time to cover the route and the standard deviation. Finally, the last three columns include information about the deviation from the nominal path during the process. Specifically, the maximum and mean deviation and the standard deviation.

Additionally, these results have been compressed in a table where the different routes’ information is merged for each maximum distance. Table 2 includes a synthesized version on the previous table with the first column indicating the maximum distance, leaving the rest of the columns to the time and deviation information, as presented in the previous table.

A close look at both tables surfaces some relevant information. Initially, in all cases, the maximum allowed deviation has been reached during the transportation process (mainly in sharp bends of the route). Therefore, users tend to use all the available space to guide the robot; it is especially notorious when users reach bends along the route. Additionally, lower mean deviation values have been observed with a maximum allowed distance of 0.250 m. However, the difference with the maximum distance 0.500 m is not that large (only 0.050 m more) based on the difference in the lane width. Finally, users can cover the path in less time with a wider lane, as they can guide the robot easily and even take shortcuts in sharp bends due to the width of the path.

As a general conclusion, the experiment subjects were able to carry out the part transportation in less time and more comfortably with a greater maximum distance *d*. Additionally, it did not impact the mean deviation value greatly as the projected arrows guide the users and allow them to re-enter the path after sharp bends or unexpected obstacles.

## 6. Conclusions and Future Work

This paper presents a novel architecture for mobile co-manipulation. The architecture includes the capability to add a path to guide the part transportation process and ensure safety by creating virtual lanes in the robot’s workspace. Additionally, the architecture incorporates a specific module to generate robot-to-human feedback to assist the process and improve the user experience in the collaborative task. Therefore, the proposed work covers all the different steps of the mobile co-manipulation for large part transportation, starting from the force-based control algorithm and finishing with the generation of suitable feedback for the system users.

The proposed approach has been implemented and tested in an industrial workshop environment, assisting operators in the transportation of large carbon fiber parts within different workstations. The obtained results show the suitability of the architecture, highlighting the utility of the robot-to-human feedback generation to guide operators along the defined safe paths of the workshop. Moreover, the addition of projected arrows helps operators to keep close to the nominal path even with wide co-manipulation lanes.

As future steps, several research paths have been identified. On the one hand, it would be interesting to investigate different cues and communication methods to improve co-manipulation tasks to create a seamless interaction between humans and robots. On the other hand, many parameters of the algorithm must be tuned manually by experts. Therefore, it would be interesting to include Artificial Intelligence techniques to extract these data from the information gathered during the co-manipulation processes as currently all this data is not further used nor exploited.

## Figures and Tables

**Table 1:** Results of the experiment.

		Time		Deviation		
d	Route	Mean	σ	Max.	Mean	σ
0.250 m	shelf to insp.	26.6 s	5.69 s	0.249 m	0.129 m	0.075 m
0.250 m	insp. to shelf	27.4 s	6.93 s	0.250 m	**0.107 m**	0.068 m
0.500 m	shelf to insp.	**23.5 s**	3.49 s	0.499 m	0.158 m	0.102 m
0.500 m	insp. to shelf	**23.2 s**	6.93 s	0.492 m	0.168 m	0.113 m

**Table 2:** Results of the experiment grouped by maximum distance.

	Time		Deviation		
d	Mean	σ	Max.	Mean	σ
0.250 m	27.0 s	5.99 s	0.250 m	**0.118 m**	0.071 m
0.500 m	**23.4 s**	3.97 s	0.499 m	0.163 m	0.108 m

## Data Availability

Experiment data is available at 10.5281/zenodo.5163197.
